# Molecular characterization of *Acinetobacter baumannii* isolated from Iraqi hospital environment

**DOI:** 10.1016/j.nmni.2017.10.010

**Published:** 2017-11-03

**Authors:** I.M.S. AL-Kadmy, A.N.M. Ali, I.M.A. Salman, S.S. Khazaal

**Affiliations:** Branch of Biotechnology, Department of Biology, College of Science, Mustansiriyah University, Baghdad, Iraq

**Keywords:** Acinetobacter baumannii, biofilm, hospital environments, multidrug-resistant biofilm

## Abstract

Healthcare-associated items are a common source of acquired infections, and hospital-acquired infections cause significant mortality and morbidity worldwide. *Acinetobacter baumannii* is the most prevalent infection-causing organism in the hospital environment. Hospital articles and objects are the main sources of infection with the ability to transmit some of the pathogenic microorganisms such as *A. baumannii*, which is considered a serious problem in therapeutic treatments. In the current study, we isolated *A. baumannii* from hospital sources and evaluated its antibiotic resistance, virulence factors and resistance gene determinants. The isolates were identified phenotypically as well as genotypically using PCR. In addition, their capability for biofilm formation and ten other virulence factors were measured. Of 112 samples, 21 showed growth of the target organism. Apart from *A. baumannii*, isolates of *Candida albicans*, *Staphylococcus* sp., *Pseudomonas aeruginosa*, *Escherichia coli* and *Klebsiella pneumoniae* were also grown. Antibiotic susceptibility test results considered all the *A. baumannii* to be multidrug-resistant isolates with the highest resistance being 100% to gentamycin, ciprofloxacin; the most effective antibiotics with 100% susceptibility was colistin and tigecycline. All *A. baumannii* isolates had MIC for ceftriaxone >32 mg/L. All *A. baumannii* isolates from the hospital environment showed multidrug resistance and had many virulence factors. They have long-term resistance to dry conditions and cause a serious public health issue.

## Introduction

Among the abundant bacterial pathogens, *Acinetobacter baumannii* is a well-known Gram-negative human pathogen in hospitals, along with *Pseudomonas aeruginosa* [1], [2]*.* The ability to gain multiple virulence factors, including resistance determinants such as serum resistance, motility, efflux pumps and iron acquisition mechanisms, help this bacterium to survive in adverse environmental conditions and facilitate the development of an infection. The ability to survive in hospital environments has helped *A. baumannii* to become one of the most successful nosocomial pathogens. It is responsible for infections in hospitalized patients as well as being non-pathogenic in healthy individuals [2]. The ability of this organism to develop multidrug resistance and to survive in hospital environments for prolonged periods has helped it to emerge as a successful opportunistic nosocomial pathogen [2]. *Acinetobacter baumannii* is widespread in clinical environments, surviving as a commensal on the skin or hair of hospital staff and patients [3] and colonizing a variety of body surfaces [4]. It is a major cause of nosocomial infections worldwide due to its remarkable propensity for the rapid acquisition of resistance to an extensive range of antimicrobial agents.

Various contaminated objects have been identified that serve as potential reservoirs for this nosocomial pathogen [3], [5]. Human utility articles, importantly computers, mouse and gloves, as well as pets, have been suggested as causes for the spread of *A. baumannii* in humans [6]. Identification of the source or reservoir of *A. baumannii* strains is very important to control future infections and to curtail ongoing outbreaks. There is evidence that in the hospital environment human infectious agents or items such as mouse, computer, or gloves might carry infectious microorganisms. The production of various virulence factors varies widely among different strains from different sources, although the cause for such variation is still unclear [7], [8]. Antibiotic resistance has also been found to play a role in virulence determination [9], [10]. The present study aimed to investigate the molecular epidemiology of *A. baumannii* isolated from items in the hospital environment and the presence of various resistance determinants to deduce their potential ability to cause human infections.

## Materials and methods

The study was conducted at Mustansiriyah University, College of Science, Department of Biology. A total of 112 samples were collected from items in the hospital environment from different Baghdad hospitals from AL-Karkh & Al-Rusafa. Each sample was washed thoroughly in PBS, and then transferred to brain–heart infusion broth for further incubation for 24 h at 37°C_._ The turbid broth of each sample specimen was inoculated onto appropriate plates, such as blood agar and MacConkey agar for standard aerobic growth, and placed at 37°C for 24 h or 48 h, respectively. The isolated organisms were identified using the automated system VITEK 2 (BioMérieux, Marcy l’Etoile, France). All the organisms identified as *A. baumannii* were used for further characterization.

### Genotype confirmation of A. baumannii by PCR

The *recA* gene (Forward CCTGAATCTTCYGGTAAAAC/Reverse GTTTCTGGGCTGCCAAACATTAC; amplified size 240 bp) and identification of the *bla*OXA-51-like gene [11], [12], [13] was used for *A. baumannii* genotypic identification. Template DNA was prepared by the boiling method as described by Ruppé et al. [14] and supernatant was used as template DNA in PCR analyses. The amplified products were further confirmed by sequencing and BLAST matching.

### Antimicrobial susceptibility testing

The MIC was found using an agar dilution method to determine the break point for *A. baumanii*, to the following antimicrobials, and disc diffusion was also performed (μg/disc) for: amikacin (30 μg), gentamicin (30 μg), imipenem (10 μg), norfloxacin (10 μg), azithromycin (15 μg), levofloxacin (5 μg) and amoxicillin + clavulanic acid (30 μg). Multidrug-resistance was defined as resistance to three or more antibiotics of the different classes. *Pseudomonas aeruginosa* ATCC 27853 and *Escherichia coli* ATCC 25922 were used as positive and negative controls, respectively (CLSI 2016). MBLs were determined by EDTA disc synergy tests using meropenem and meropenem plus EDTA and the modified Hodge method. For calculation, intermediate isolates were considered as resistant.

### Molecular characterization of antibiotic resistant determinants

To detect resistance determinants as given in Table 1, chromosomal DNA was extracted from the isolates and 5 μL of this extract was used for PCR assay to amplify resistance determinants using primers and conditions of amplification as previously reported [11], [15], [16], [17], [18], [19], [20], [21], [22]. Amplified products were subjected to sequencing at Microgen (Seoul, South Korea). The PCR products were analysed in horizontal electrophoresis using a 1% agarose gel and 10-kb DNA ladder (Kapa Biosystem, Cape Town, South Africa) as a molecular marker, then PCR products were visualized by UV light at 336 nm.

**Table 1:** Primers details used in this study

Primer name	Primer sequence	Base length	References
CTX-M1C-f	GACTATTCATGTTGTTGTTATTTC	923	[15]
CTX-M1C-R	TTACAAACCGTTGGTGACG		
preOXA-48-f	TATATTGCATTAAGCAAGGG	800	[16]
preOXA-48-r	CACACAAATACGCGCTAACC		
VIM2-f	GATGGTGTTTGGTCGCATA	390	[17]
VIM2-r	CGAATGCGCAGCACCAG		
SHV-1	CGCCGGGTTATTCTTATTTGTCGC	1016	[17]
SHV-2	TCTTTCCGATGCCGCCGCCAGTCA		
CTX-M2A	ACTCAGAGCATTCGCCGCTCA	879	[18]
CTX-M2B	TTATTGCATCAGAAACCGTG		
CTX-M9A	ATGGTGACAAAGAGAGTGCAACG	837	[18]
CTX-M9B	ACAGCCCTTCGGCGATGATTC		
IMP2-f	GGAATAGAGTGGCTTAAYTCTC	232	[19]
IMP2-r	CCAAACYACTASGTTATCT		
TEM-1	ATAAAATTCTTGAAGAC	1079	[20]
TEM-2	TTACCAATGCTTAATCA		
preNDM-f	CACCTCATGTTTGAATTCGCC	984	[21]
preNDM-r	CTCTGTCACATCGAAATCGC		
Bla KPC	ATGTCACTGTATCGCCGTCT	882	[22]
Bla KPC	TTACTGCCCGTTGACGCCC		
*cnf1-F*	AAGATGGAGTTTCCTATGCAGGAG	498	[23]
*cnf1-R*	CATTCAGAGTCCTGCCCTCATTATT		
*csgA-F*	ACTCTGACTTGACTATTACC	200	[23]
*csgA-R*	AGATGCAGTCTGGTCAAC		
*cvaC-F*	CACACACAAACGGGAGCTGTT	680	[23]
*cvaR-F*	CTTCCCGCAGCATAGTTCCAT		
*iutA-F*	GGCTGGACATCATGGGAACTGG	300	[23]
*iutA-R*	CGTCGGGAACGGGTAGAATCG		

### Virulence factor detection assays

Phenotypic detection of various virulence factors of *A. baumannii* isolates was performed to detect their biofilm formation, *N*-acyl-homoserine lactone (AHL) production, quorum-sensing synthase molecule *abaI*, serum resistance and PCR detection of putative virulence genes *cnf1*, *csgA*, *cvaC* and *iutA* following published protocols from literature as given below [23] (Table 1).

### Biofilm formation

Biofilm production on an abiotic surface was quantified as previously described [11]. In brief, 5 mL overnight cultures of *A. baumannii* grown at 37°C were diluted to an optical density at 600 nm (OD_600_) of 0.003 in Luria–Bertani medium and 500-μL aliquots were dispensed into polystyrene tubes in triplicates. Following 24 h of incubation at 37°C, the medium was removed and the tubes were washed gently once with deionized water and stained with 1% (weight/volume) crystal violet. After washing three times with distilled water, the adherent bacteria stained with crystal violet were dissolved in 1 mL of 100% methanol and quantified by measuring OD_540_ [11].

### PCR-based detection of virulence genes

The virulence genes *cnf1*, *csgA*, *cvaC*, *iutA* were amplified as described previously [23]. The list of primers and PCR conditions are included in Table 1. The cycling conditions were as follows: one cycle 95°C for 4 min; 30 cycles 95°C for 50 s, 58°C for 60 s, 72°C for 45 s; one cycle 72°C for 8 min.

### Serum resistance assay

Serum resistance was assessed according to the protocol of Kim et al. [24]. Briefly, bacterial cells grown in TSBD medium for 14 h were washed and re-suspended in PBS to an OD_600_ of 1.0. Human serum from five healthy individuals was pooled and diluted in PBS to a 40% final dilution. Heat-inactivated serum was prepared by incubating the same serum at 56°C for 30 min. Bacterial suspensions were then added to human serum or heat-inactivated serum to obtain a bacterial cell concentration of 1 × 10^7^ CFU/mL and samples were incubated at 37°C for 2 h. Viable counts were determined at 0-h and 2-h time-points in triplicates.

### AHL production

Production of AHL was detected among the 21 isolates as described previously by Kumar et al. [24]. In brief, reporter strain *E. coli* MG4 (pKDT17) (ampicillin 100 mg/L), was used for the detection and measurement of AHLs, and AHL biosensors *Chromobacterium violaceum* CV026 (Luria–Bertani broth, kanamycin 20 mg/L). AHL was detected in Luria agar plates covered with 40 μL of X-Gal (20 mg/mL) by streaking 1 cm apart with reporter strains *E. coli* MG4 or PAO-JP2 and the strain to be tested. AHLs produced by the organisms, diffused through the agar resulting in blue colour in the reporter strain. AHLs were then extracted with equal volumes of acidified ethyl acetate from the overgrown cultures and then dried over magnesium sulphate. The extracts were resuspended in HPLC grade ethyl acetate and evaluated by thin-layer chromatography as described by Shaw et al. [25].

### AHL quantification

Quantification of AHL was performed as described by Miller [26]. In brief, the supernatant culture was extracted from overnight-grown culture for β-galactosidase activity and the reporter strain was diluted 1:1 in Z buffer and assayed for β-galactosidase activity by using *o*-nitrophenyl-d-galactopyranoside as a substrate [26].

### Detection of abaI gene producing the QS signal molecules

The *abaI* gene encoding an autoinducer synthase was identified by PCR using already published primers [27] Forward 5′-GTACAGTCGACGTATTTGTTGAATATTTGGG-3′ and Reverse 5′-CGTACGTCTAGAGTAATGAGTTGTTTTGCGCC-3′ were used with the following PCR cycling parameters: initial denaturation at 94°C for 10 min followed by 30 cycles of denaturation at 94°C for 30 s, Primer annealing at 66.5°C for 30 s, primer extension at 72°C for 1 min and the final extension at 72°C for 5 min. The resulting PCR amplicon was visualized in a trans illuminator [27].

## Results

**Fig. 1:**
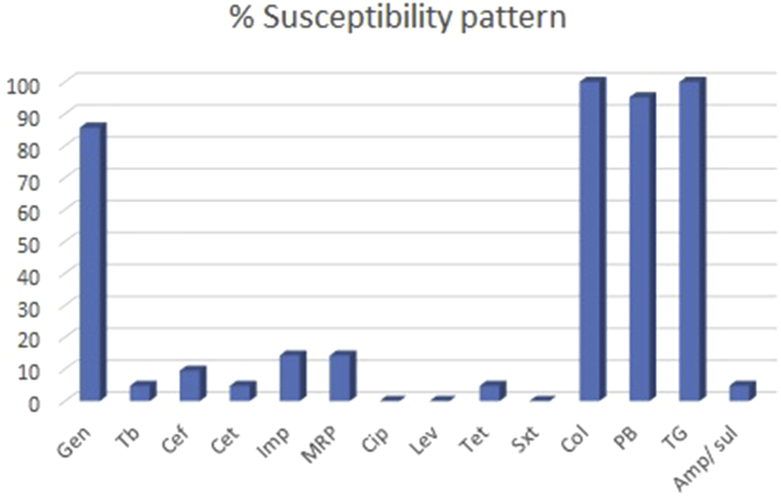
Antibiotic susceptibility profile for study isolates.

**Fig. 2:**
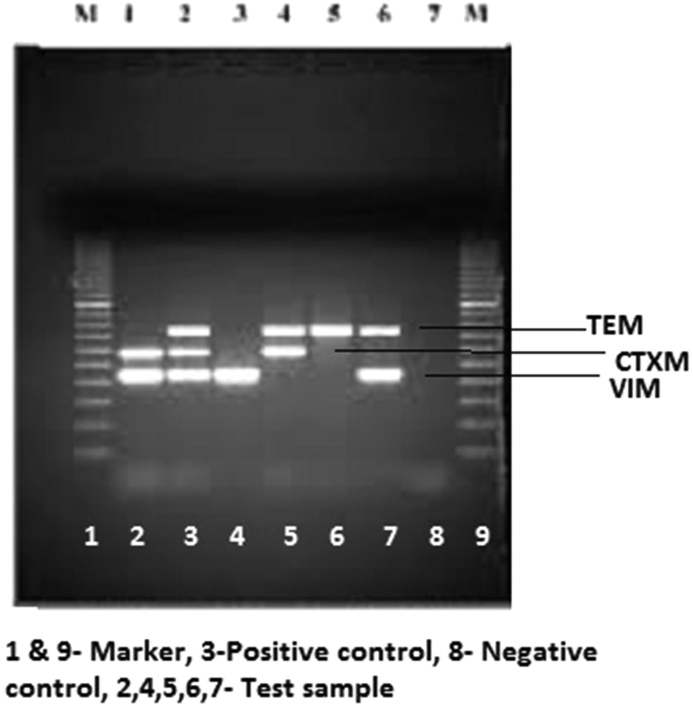
Gel picture for PCR used in this study.

During the study period, a total of 112 human objects from the hospital environment were collected, from which 21 samples were grown and identified as *A. baumanii*. All *A*. *baumannii* isolates were confirmed by amplification of *recA* and *bla*_*OXA-51*_*-*like gene. The isolates were 100% resistant to ciprofloxacin, levofloxacin and trimethoprim-sulfamethoxazole. More than 90% resistance was seen to tobramycin, cefepime, ceftriaxone, tetracycline and β-lactams. Resistance to meropenem and imipenem was close to 86% (Table 2, Fig. 1). All isolates were susceptible to colistin and tigecycline and <6% were resistant to polymyxin B (Table 2). MIC_50_ and MIC_90_ values are shown in Table 3. MIC for ceftriaxone was >32 mg/L for all the isolates (Table 3). Most isolates were positive for CTMX and TEM genes, VIM and SHV were present in four and three isolates, respectively, and only one was harbouring IMP. None of isolated strains had OXA or NDM (Fig. 2).

**Table 2:** Antibiotic susceptibility profile of study isolates

Sample ID	Organism	Antibiotic susceptibility profile														Ceftriaxone MIC (E-test)	PCR results (ESBL)
		Gen	Tb	Cef	Cet	Imp	MRP	Cip	Lev	Tet	Sxt	Col	PB	TG	Amp/sul		
1	*A.baumannii*	S	S	R	R	R	R	R	R	R	R	S	S	S	R	>32 mg/L	CTXM, TEM
2	*A.baumannii*	R	R	R	R	R	R	R	R	R	R	S	S	S	R	>32 mg/L	CTXM
3	*A.baumannii*	R	R	R	R	R	R	R	R	R	R	S	S	S	R	>32 mg/L	CTXM
4	*A.baumannii*	R	R	R	R	R	R	R	R	R	R	S	R	S	R	>32 mg/L	TEM, SHV, IMP
5	*A.baumannii*	S	R	R	R	R	R	R	R	R	R	S	S	S	R	>32 mg/L	TEM, SHV
6	*A.baumannii*	S	R	R	R	R	R	R	R	R	R	S	S	S	R	>32 mg/L	CTXM, TEM
7	*A.baumannii*	S	R	R	R	R	R	R	R	R	R	S	S	S	R	>32 mg/L	TEM
8	*A.baumannii*	S	R	R	R	S	S	R	R	R	R	S	S	S	R	>32 mg/L	CTXM, TEM
9	*A.baumannii*	S	R	R	R	R	R	R	R	R	R	S	S	S	R	>32 mg/L	CTXM
10	*A.baumannii*	S	R	S	R	S	S	R	R	R	R	S	S	S	R	>32 mg/L	NEG
11	*A.baumannii*	S	R	R	R	R	R	R	R	R	R	S	S	S	R	>32 mg/L	CTXM, TEM
12	*A.baumannii*	S	R	S	S	S	S	R	R	S	R	S	S	S	S	>32 mg/L	NEG
13	*A.baumannii*	S	R	R	R	R	R	R	R	R	R	S	S	S	R	>32 mg/L	CTXM, TEM
14	*A.baumannii*	S	R	R	R	R	R	R	R	R	R	S	S	S	R	>32 mg/L	CTXM, TEM
15	*A.baumannii*	S	R	R	R	R	R	R	R	R	R	S	S	S	R	>32 mg/L	CTXM, TEM
16	*A.baumannii*	S	R	R	R	R	R	R	R	R	R	S	S	S	R	>32 mg/L	CTXM, SHV
17	*A.baumannii*	S	R	R	R	R	R	R	R	R	R	S	S	S	R	>32 mg/L	CTXM, TEM
18	*A.baumannii*	S	R	R	R	R	R	R	R	R	R	S	S	S	R	>32 mg/L	CTXM, TEM
19	*A.baumannii*	S	R	R	R	R	R	R	R	R	R	S	S	S	R	>32 mg/L	CTXM, TEM
20	*A.baumannii*	S	R	R	R	R	R	R	R	R	R	S	S	S	R	>32 mg/L	CTXM, TEM
21	*A.baumannii*	S	R	R	R	R	R	R	R	R	R	S	S	S	R	>32 mg/L	CTXM, TEM
	Susceptible	18	1	2	1	3	3	0	0	1	0	21	20	21	1		
	Resistant	3	20	19	20	18	18	21	21	20	21	0	1	0	20		

Abbreviations: Amp/sul, ampicillin/sulbactam; Cef, cefepime; Cet, ceftriaxone; Cip, ciprofloxacin; Col, colistin; Gen, gentamicin; Imp, imipenem; Lev, levofloxacin; MRP, meropenem; Sxt, trimethoprim/sulfamethoxazole; PB, polmyxin B; TB, tobramycin; Tet, tetracycline; TG, tigecycline.

**Table 3:** Antibiotic susceptibility profile of study isolates with MIC_50_ and MIC_90_ value

Antibiotic family	Antibiotic	Breakpoints			MIC_50_ (mg/L)	MIC_90_ (mg/L)	Resistant (%)	Susceptible (%)
		S	I	R				
Aminoglycosides	Gentamicin	≤4	8	≥16	4	8	14.3	85.7
	Tobramycin	≤4	8	≥16	16	32	95.2	4.8
Cephalosporins	Cefepime	≤8	16	≥32	16	32	90.5	9.5
	Ceftriaxone	≤8	16–32	≥64	16	32	95.2	4.8
Carbapenems	Imipenem	≤2	4	≥8	8	16	85.7	14.3
	Meropenem	≤2	4	≥8	8	16	85.7	14.3
Fluoroquinolones	Ciprofloxacin	≤1	2	≥4	4	8	100.0	0.0
	Levofloxacin	≤2	4	≥8	8	16	100.0	0.0
Tertracycline	Tertracycline	≤4	8	≥16	8	16	95.2	4.8
Trimethoprim/sulfamethoxazole	Trimethoprim/sulfamethoxazole	≤2/38	NA	≥4/76	NA	≥4/76	100.0	0.0
Fluoroquinolones	Colistin	≤2	NA	≥4	0.5	1	0.0	100.0
	Polmyxin B	≤2	NA	≥4	0.5	1	4.8	95.2
Glycylcyclin	Tigecycline	≤2	4	≥8	0.5	1	0.0	100.0
β-lactam/β -lactamase inhibitor combinations	Ampicillin/sulbactam	≤8/4	16/8	≥32/16	NA	≥32/16	95.2	4.8

### Biofilm production

**Fig. 3:**
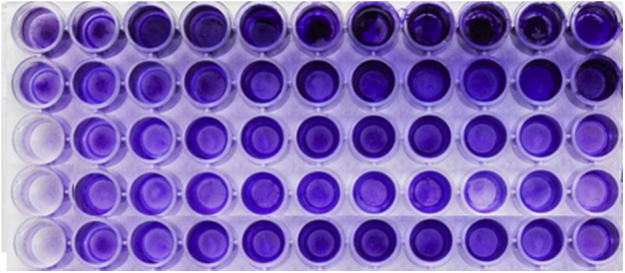
Biofilm formation in microtiter plate.

The ability of the 21 *A. baumannii* isolates to form biofilms was investigated and biofilm formation was compared among the different *A. baumanii* strains after incubation in polystyrene microtitre wells (Fig. 3). All of the clinical isolates (21/21) formed strong biofilms on abiotic surfaces. There were no weak biofilm producers in our observations.

### Resistance to human serum

The 21 *A. baumannii* strains were compared for their ability to survive the bactericidal activity of human serum. Overall, the 21 strains displayed a comparable resistance to human serum whereas the ATCC 17978 strain tested showed very low survival in the presence of human serum.

### AHL production

**Fig. 4:**
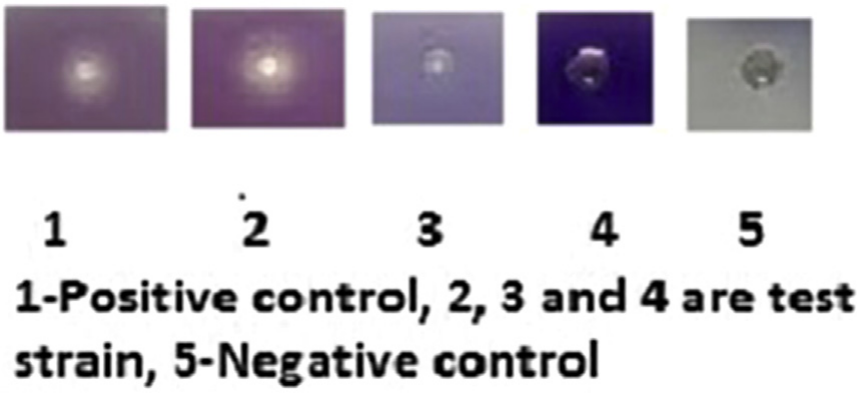
Production of AHLs for study isolates.

All these isolates were also screened for the AHL production and 14 effectively produced AHL molecules by using the CV026 biosensor monitor system. On analysis of the biofilm-forming capabilities of the 14 isolates that produced the AHLs, they all formed strong biofilms. In this study, it has been shown that among the 21 biofilm-forming isolates, only 14 produced AHLs (Fig. 4).

### PCR-based detection of virulence genes and QS-producing abaI

Table 4 shows the distribution of the putative virulence genes among the 21 *A. baumannii* strains isolated in this study. Overall, *csga* (14/21) and *cnf1* (10/21) were the most commonly detected virulence genes. C*vaC* and *iutA* genes were identified in 2/21 and 5/21 strains, respectively.

**Table 4:** PCR-based detection of virulence genes and QS producing *abaI*

Sample/S.No	Biofilm	AHL production	Serum resitance	*Cnf+*	*CsgA*	*CvaC*	*IutA*	*abaI*
1	+	+	+	+	+	−	+	+
2	+	−	+	+	−	−	−	−
3	+	+	+	−	+	+	+	+
4	+	+	+	+	+	−	−	+
5	+	−	+	−	−	−	−	−
6	+	−	+	+	+	−	−	−
7	+	+	+	−	+	−	−	+
8	+	+	+	+	−	−	−	+
9	+	+	+	−	+	+	+	+
10	+	−	+	+	+	−	−	−
11	+	+	+	+	+	−	−	+
12	+	+	+	+	−	−	−	+
13	+	+	+	−	+	−	−	+
14	+	−	+	−	−	−	−	−
15	+	−	+	+	+	−	+	−
16	+	+	+	−	+	−	−	+
17	+	+	+	−	+	−	−	+
18	+	+	+	−	−	−	−	+
19	+	−	+	−	+	−	−	−
20	+	+	+	+	+	−	+	+
21	+	+	+	−	−	−	−	+

### Detection of abaI gene producing the QS signal molecules

PCR for the *abaI* gene produced amplicons of 382 bp in 14/21 isolates that produced the QS signal molecules.

## Discussion

*Acinetobacter baumannii* is widespread in clinical environments, surviving as commensals on the skin of hospital staff and patients, being especially concentrated in moist regions such as axilla, groin and fingertips of personnel [1], [3]. The current study proved that items in the hospital environment were sources for infections. The ability to survive in hospital environments has assisted *A. baumannii* to emerge as one of the most successful nosocomial pathogens and enhanced its capability to become the leading pathogen in intensive care units due to its increasing multidrug resistance. *Acinetobacter baumannii* has become a threat in hospitals with its rapid emergence as a resistant organism through antibiotic pressure. It grows as a nosocomial because it can develop and survive in hospital environments for prolonged periods as a result of its exceptional ability to produce highly resistant biofilms [2], [4].

Positive culture results from hospital samples showed the diversity of bacterial isolates: two *Candida albicans*, 11 *Staphylococcus* sp., five *Pseudomonas aeruginosa* and one isolate of *Klebsiella pneumoniae* were also recovered. This study found that 21 samples showed the presence of *A. baumannii*, more than any other organism isolated. The samples were sourced from commonly used items in the hospitals, which provide a route for the spread of infections to various categories of hospital staff. The results of antibiotic susceptibility testing showed a high level of resistance against most antibiotics, making it a multidrug-resistant bacterium.

Though initially considered to be an organism of low virulence, *A. baumannii* is gradually gaining virulence [8], [9], [10] and this study revealed that each isolate had more than one virulence factor (Table 4). All the isolates had biofilm forming ability and were negative in gelatinase and protease reactions. Most isolates were capsulated and found to be positive in twitching motility and as pellicle producers. These virulence factors play a significant role in resistance to defence mechanisms. Moreover, *A. baumannii* is able to cause a wide range of infections, is increasingly resistant to antibiotics and may be associated with high mortality rates in some patients. Its virulence factors and pathogenicity mechanisms were largely unknown, and genetics study are still limited for this bacterium.

In this study we showed 100% of our isolates producing biofilms. All strains were screened for AHL QS signal molecule production but only 66% of strains effectively produced AHL molecules by using the CV026 biosensor monitor system. Identification of AHLs in *A. baumannii* is important due to increasing nosocomial infections and because the QS cascades are possible drug target factors for future therapies to combat *A. baumannii* infections^.^ We showed that among the 21 biofilm-forming isolates, only 14 produced AHLs, which may be because biofilm formation is a multifactorial event involving a number of molecules in which QS signals are one of the factors [27]. We also tried to identify the types of AHLs produced using TLC and two biosensor strains, *C. violaceum* CV026 and *Agrobacterium tumefaciens* A136. *Chromobacterium violaceum* strain CV026 is a violacein and AHL-negative double mini Tn5 mutant strain with transposons inserted into the *CviI* AHL synthase gene and violacein repressor gene. This strain produces the pigment violacein by using the exogenous AHL and detects C4 to C8-homoserine lactones (HSLs) but most strongly C6-HSL [28]. All the 14 strains that produced AHLs were found to be C6-HSL type by development with the biosensor *C. violaceum* CV026. Even though the biosensor strain used in this study detects a wide range of exogenous AHLs (C4- to C8-HSLs) we identified only the C6-HSL type of AHL in all 14 isolates. The negative results may be due to no/low production of AHL signals by these strains. This method of screening is rapid and easy to perform in clinical laboratories. Moreover, this method can be used to screen strains for AHL production directly from patient samples. Quantification of β-galactosidase activity showed production of variable levels of AHLs by *A. baumannii*, indicating the difference in source and amounts of AHLs, which may be due to differences in AHL production between strains. We also identified an autoinducer synthase gene in *A. baumanii*, similar to the members of the LuxI family, designated as *abaI* among the 21 *A. baumanii* strains. But *abaI* was identified in only 66% of *A. baumanii* that produced the AHLs phenotypically. This approach can be exploited for detection of QS signal molecules directly from patient samples with the presence of pathogen [28].

*Csga*, *cnf1*, *cvaC* and *iutA* virulence genes were prevalent among the *A. baumannii* strains of our clinical infections. Overall, *csga*, *cnf1*, *cvaC* and *iutA* virulence genes were detected in 66.7%, 47.6%, 9.5% and 23.8% of strains respectively. Previous reports demonstrated total prevalence of *csga*, *cnf1*, *cvaC* and *iutA* virulence genes among urinary tract infection samples to be 55%, 40%, 10% and 30%, respectively, which is similar to our results. In contrast, Momtaz et al. [29] reported that the prevalences of *csga*, *cnf1*, *cvaC* and *iutA* virulence genes from Iran were 12.39%, 35.53%, 21.48% and 19%, respectively, which was slightly lower than our results. These genes are the most common causes of adhesion and invasion of *A. baumanni* to the epithelial cells of the human organs [23].

In conclusion, we identified highly resistant and virulent strains of *A. baumannii* from the hospital environment in different hospitals in Baghdad. We observed strains with multifactorial phenotypes related to pathogenesis, such as biofilm formation, motility and resistance to human serum, suggesting the ability to persist, which accounts for *A. baumannii* survival in the hospital setting and, ultimately, for its ability to cause outbreaks of infection [8].

The most commonly detected virulence genes were *csga* and *cnf1*. The result of this study also found evidence for antibiotic resistance mediated through various genes in *A. baumannii* from hospital environments. Rational use of antibiotics according to the results of disc diffusion studies can help to decrease the prevalence of resistance [23]. Studies on identification of resistance gene transfer through plasmids or insertion elements, which can further deduce their probable mode of transfer [30], is required. Such information may help us to control and eradicate persistent endemic and epidemic clones of *A. baumannii*. In addition, rigorous infection control measures including defensive measures to prevent the propagation of plasmid resistance determinants in environments, can prevent further spread of resistance.

## Conflict of Interest

Authors have nothing to disclose.
